# Differences in the development of autogenous nerves between the upper and lower urinary tract

**DOI:** 10.3892/etm.2013.888

**Published:** 2013-01-07

**Authors:** QIMIN CHEN, JINQUAN CAI, CHENGREN SHI, JIE SUN, MINZHI YIN, PING SHEN

**Affiliations:** 1 Department of Urology, Shanghai Children’s Medical Center affiliated to Shanghai Jiao Tong University School of Medicine, Shanghai 200127;; 2 Department of Urology, Fuzhou General Hospital, Nanjing Command, PLA, Fuzhou 350025;; 3 Department of Pediatric Surgery, Xinhua Hospital, Shanghai 200092;; 4 Department of Pathology, Shanghai Children’s Medical Center affiliated to Shanghai Jiao Tong University School of Medicine, Shanghai 200127, P.R. China

**Keywords:** urinary tract, nervous plexus, neuroganglion, development

## Abstract

The aim of this study was to observe the development and distribution of autogenous nerves in the urinary tract of New Zealand rabbits. Animals of various ages were used in this study, including 2, 3 and 3.5 weeks (gestational ages) and 1, 4, 8 and 12 weeks (postnatal). Samples were selected at various sites of the urinary tract. Immunohistochemical staining methods were used to investigate the nervous plexus and neuroganglia on the wall of the urinary tract. Myenteric plexuses and ganglia first appeared at the gestational age of 3 weeks. They decreased in the pelvis and ureter as the animals developed, until at the end of postnatal week 8, all nerves disappeared completely. However, nerves existed in the bladder and posterior urethra permanently. The development and distribution of myenteric nerves is different between the upper and lower part of the urinary tract. Our study aimed to investigate this further.

## Introduction

Nerve fibers are distributed in the submucosa and myenteric structure of normal intestines, which are called the Meissner’s plexus and Auerbach’s plexus, respectively (1). The nervous plexus contains mature neuroganglion cells which maintain intestinal peristalsis to transport food (2). Without neuroganglion cells, preganglionic fibers of the parasympathetic ganglion from sacral spinal cord segments S2–S4 are not able to form synapses and generate postganglionic fibers in the intestinal tract, which causes preganglionic nerve fiber hyperplasia and incoordination of intestinal systaltic and peristaltic movement, resulting in difficulties in food transportation (3).

Urine experiences a similar sequence of migration from production in the kidneys to secretion through the urethra. The structures participating in urine transportation, including the kidneys, ureter, bladder and urethra, are collectively called the urinary tract. The urinary tract is composed of mucosa, myometrium and an outer membrane (4). To date, there is insufficient evidence on whether nerve fibers are distributed in the urinary tract and whether there is a correlation between nerve fiber activity and urine transportation. However, a knowledge of nerve development and distribution in the urinary tract is important for disease diagnosis and treatment. Therefore, we designed the current study to observe the development and distribution of autogenous nerves in the urinary tract of New Zealand rabbits of varying ages through section analysis.

## Materials and methods

### Animals

Twenty New Zealand rabbits were selected aged 1, 4, 8 and 12 weeks. They were sacrificed by air injection in the ear marginal and the urinary system, including the kidneys, ureter, bladder and posterior urethra, was removed.

A further three pregnant rabbits were selected at 2, 3 and 3.5 weeks of gestation. They were sacrificed by air injection in the marginal ear vein. Five fetal rabbits of each gestational age were obtained by cesarean section. The fetal rabbits were dissected and embryonic tissues, including the kidneys, ureter, bladder and posterior urethra, were removed.

### Immunohistochemistry

All organs were fixed in 0.4% formalin. Samples of each urinary organ were taken as follows: i) kidney: parallel to the renal pelvis; ii) ureter: upper-third of the ureter and lower-third of the ureter; iii) bladder: upper half of the bladder and lower half of the bladder including the trigone of the bladder; iv) posterior urethra: proximal to the pubic symphysis.

Samples were embedded in paraffin and sections were cut. Hematoxylin and eosin (H&E), protein gene product 9.5 (PGP9.5) and neuron-specific enolase (NSE) staining methods were used following manufacturer’s instructions. All kits used were purchased from Shanghai Changdao Biotech Co. Ltd. (Shanghai, China).

## Results

The H&E stained urinary organs were barely visible to the naked eye and only a vague structure could be observed microscopically at a gestational age of 2 weeks. At gestational ages of 3 and 3.5 weeks, urinary organs began to form. The glomeruli were observed to be immaturely developed and the urinary tract was beginning to form.

**Figure 1.**
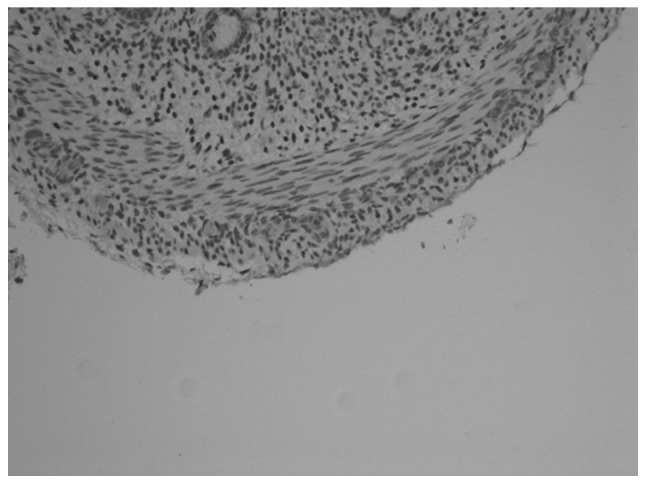
Positive result of immunohistochemical staining for NSE in the myenteric plexus of the ureter at the gestational age of 3 weeks (magnification, ×200). NSE, neuron-specific enolase.

**Figure 2.**
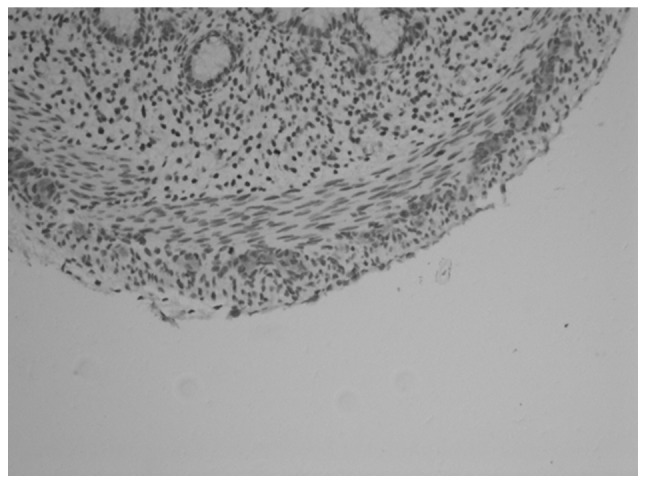
Positive result of immunohistochemical staining for PGP9.5 in the myenteric plexus of the ureter at the gestational age of 3 weeks (magnification, ×200). PGP9.5, protein gene product 9.5.

**Figure 3.**
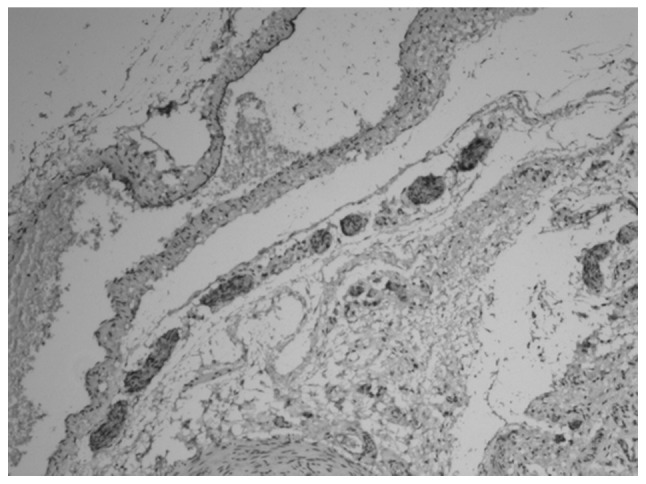
Positive result of immunohistochemical staining for NSE in the outer membrane nervous plexus of the pelvis at the age of 8 weeks (magnification, ×200). NSE, neuron-specific enolase.

**Figure 4.**
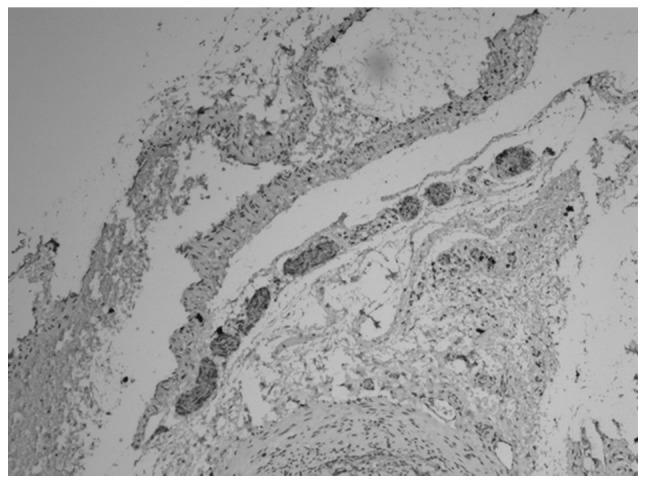
Positive result of immunohistochemical staining for PGP9.5 in the outer membrane nervous plexus of the pelvis at the age of 8 weeks (magnification, ×200). PGP9.5, protein gene product 9.5.

**Figure 5.**
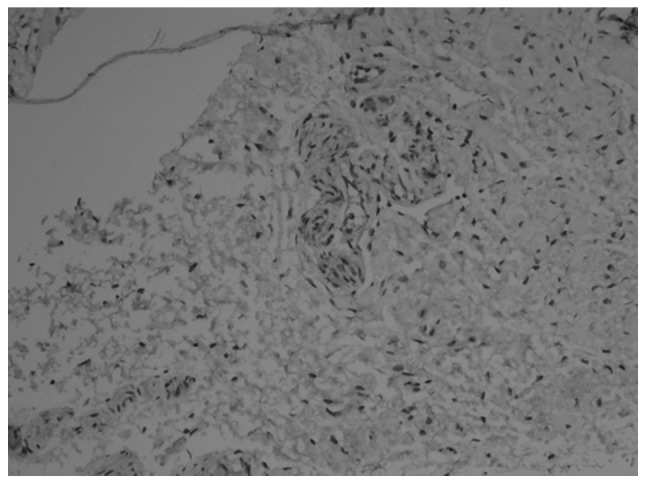
Positive result of immunohistochemical staining for NSE in the myenteric plexus of the urethra at the age of 12 weeks (magnification, ×200). NSE, neuron-specific enolase.

**Figure 6.**
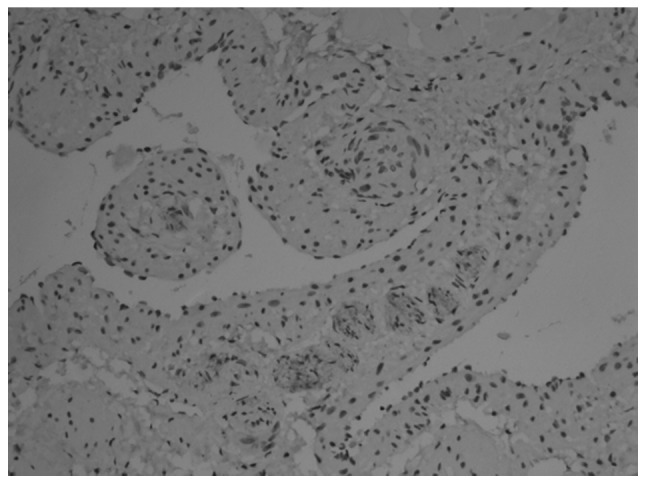
Positive result of immunohistochemical staining for PGP9.5 in the myenteric nervous plexus of the urethra at the age of 12 weeks (magnification, ×200). PGP9.5, protein gene product 9.5.

PGP9.5 and NSE revealed similar staining results. In animals at gestational ages of 3 and 3.5 weeks, the myenteric plexus and outer membrane of the nervous plexus were present in the renal pelvis to the posterior urethra and neuroganglion cells were sparsely distributed (Figs. 1 and 2). Animals aged 1 week demonstrated sparse myenteric plexus distribution in the tissue from the renal pelvis to the ureter and neuroganglion cells disappeared. At 8 weeks, the myenteric plexus completely disappeared; however, plexus remained at the site of the outer membrane (Figs. 3 and 4). At 12 weeks, the myenteric plexus showed clear, positive staining in the tissue from the bladder to the posterior urethra (Figs. 5 and 6) and the outer membrane of the nervous plexus existed permanently.

## Discussion

The main function of the urinary system is to generate, transport and excrete urine through the urinary tract. To a degree, difficulty in urine transportation is the main cause of functional urinary obstruction (5). A number of organic diseases presenting obstruction in the renal pelvis, ureter or posterior urethral valve may cause further difficulties in urine transportation (6). Primary and secondary urine transportation irregularities may influence nerve fiber development and distribution (7). Under normal conditions, the neural regulation of the urinary system is complicated. The sympathetic and parasympathetic nerves are the principal fibers that control urinary function. Certain somatic nerves participate in controlling bladder and urethral function, urine reservoir filling and urination (8). Studies into nerve development in the urinary tract and its function in the process of urine formation and transportation are lacking. However, it is important to understand the nerve distribution in the urinary organs to comprehend the etiology and pathology of diseases.

Nerve fibers develop during the embryonic phase. Obstacles to embryonic nervous development influence the function of relative organs (9–12). In the sixth week of gestation in the development of the human digestive tract, the neuroblasts in the neural crest move downwards in the digestive tract wall to form neuroganglion cells of the myenteric plexus. The myenteric neuroganglion cells continue to move and form submucosal neuroganglion cells. This movement is complete by the twelfth week of embryonic development (13).

Compared with research on the digestive tract, studies concerning nerve development in the urinary tract are scarce. In the current study, we measured the nerve distribution in the urinary tract wall of New Zealand rabbits at various gestational ages, by immunohistochemistry. The distribution of the nerve plexus and neuroganglion of the outer membrane may be the residual of peripheral nerves, therefore the myenteric plexus represents the autogenous nerve network of the urinary organs. Our study revealed that in the urinary system of New Zealand rabbits, the myenteric plexus of the upper urinary organs is formed when the kidneys are in embryonic form. As the embryo develops, the nerve plexus starts to degenerate and completely disappears in the adult phase. The myenteric plexus of the lower urinary organs is present continuously from a gestational age of 3 weeks to the adult phase, and its neuroganglion cells are also present. This demonstrates that the autogenous nerve development and distribution of urinary organs is completely different from that of the digestive tract. Additionally, the autogenous nerve development of the upper and lower urinary organs are also different.

In the embryo, the upper and lower urinary tract have different formation processes. In the fourth week of human embryonic development, the ureteric bud protrudes from the mesonephric duct and grows into renal blastema. In the fifth week, it forms the renal pelvis (14). The bladder originates from cloaca. During weeks 4–6 of human embryonic development, the cloaca is divided into two parts by the urogenital diaphragm. The ventral part grows into the bladder and proximal urethra and the dorsal part grows into the hindgut (15). We consider that different origins of fetation result in the contrasting nerve distribution in the upper and lower urinary tract. Judging from the development period, the shaping of the urogenital sinus occurs earlier than the development of intestinal neurons. Autogenous nerve development in the bladder and posterior urethra from the cloaca has a close correlation with the digestive tract so the distribution of the myenteric plexus in the urinary system may be similar to that in the intestines.

The myenteric plexus may be involved in the functioning of the lower urinary organs. Physiological observations indicate that the bladder and posterior urethra may actively control urine transportation (16,17). Contraction of the bladder and the opening of the posterior urethra are synchronized to guarantee normal urination (18). In urine reservoir filling and micturition, myenteric nerve fibers regulate and coordinate the process (19,20). The renal pelvis and ureter are not actively involved in urine transportation and their autogenous nerve develops weakly and gradually degenerates after birth. In the process of urine transportation, signaling in the muscle and tissues ensure the normal systaltic and peristaltic action of the organs (21). The uriniferous tubule of the renal parenchyma is the site of fluid excretion and absorption (22). A concentration change in water-electrolyte levels in the microenvironment affects the amount of urine and its composition. Therefore, the humoral regulation is more important and almost has no autogenous nerve distribution.

Autogenous nerve development and distribution in the urinary tract is an important subject area. Our study has shown that nerve development and distribution in the upper and lower urinary tract are different, therefore it is difficult to clearly explain the correlation between urination tract nerve distribution and disease. This research is still in the early stages and further research is required.
